# Highly Pathogenic Avian Influenza (HPAI H5Nx, Clade 2.3.4.4.b) in Poultry and Wild Birds in Sweden: Synopsis of the 2020–2021 Season

**DOI:** 10.3390/vetsci9070344

**Published:** 2022-07-08

**Authors:** Malin Grant, Caroline Bröjer, Siamak Zohari, Maria Nöremark, Henrik Uhlhorn, Désirée S. Jansson

**Affiliations:** 1 Department of Disease Control and Epidemiology, National Veterinary Institute, 751 89 Uppsala, Sweden; malin.grant@sva.se (M.G.); maria.noremark@sva.se (M.N.); 2 Department of Clinical Sciences, Swedish University of Agricultural Sciences, 750 07 Uppsala, Sweden; 3 Department of Pathology and Wildlife Diseases, National Veterinary Institute, 751 89 Uppsala, Sweden; caroline.brojer@sva.se (C.B.); henrik.uhlhorn@sva.se (H.U.); 4 Department of Microbiology, National Veterinary Institute, 751 89 Uppsala, Sweden; siamak.zohari@sva.se; 5 Department of Animal Health and Antimicrobial Strategies, National Veterinary Institute, 751 89 Uppsala, Sweden

**Keywords:** AIV, avian influenza virus, epidemiology, phylogeny, poultry, surveillance, whole-genome sequencing, wild birds

## Abstract

**Simple Summary:**

Highly pathogenic avian influenza is a virus-induced contagious disease that has killed a large number of poultry and wild birds in Europe in the recent decade and is an increasing problem worldwide. In the winter of 2020–2021, Sweden experienced its worst period to date when the disease was diagnosed on 15 commercial poultry farms and over 2.2 million birds died or were euthanised. The disease was also diagnosed in 130 wild birds and nine flocks of hobby, game or zoo birds between 1 October 2020 and 30 September 2021. The aim of this article was to describe the influenza situation in Sweden during this period and to add to the knowledge related to the alarming situation with highly pathogenic influenza in birds. The disease caused animal suffering and death in wild and domestic birds and incurred high costs due to losses and extensive measures to stop spread. The outbreak investigations, where contacts were traced and virus strains were compared, concluded that the virus was brought to poultry farms by wild birds in most cases. More research is needed to obtain knowledge on risk factors, biosecurity, and wild bird presence on poultry farms to prevent future disease outbreaks.

**Abstract:**

Highly pathogenic avian influenza (HPAI, Gs/Gd lineage) was introduced to Europe in 2005 and has since caused numerous outbreaks in birds. The 2020–2021 season was the hitherto most devastating when considering bird numbers and duration in Europe. Surveillance data, virologic results and epidemiologic investigations from the 2020–2021 outbreaks in Sweden were analysed. Subtypes H5N8 and H5N5 were detected on 24 farms with poultry or other captive birds. In wild birds, subtypes H5N8, H5N5, H5N1, H5N4, H5Nx were detected in 130 out of 811 sampled birds. There was a spatiotemporal association between cases in wild birds and poultry. Based on phylogeny and epidemiology, most of the introductions of HPAI to commercial poultry were likely a result of indirect contact with wild birds. A definite route of introduction to poultry could not be established although some biosecurity breaches were observed. No spread between farms was identified but airborne spread between flocks on the same farm was suspected. Our findings exemplify the challenges posed by the continuously changing influenza viruses that seem to adapt to a broader species spectrum. This points to the importance of wild bird surveillance, compliance to biosecurity, and identification of risk factors for introduction on poultry farms.

## 1. Introduction

Highly pathogenic avian influenza (HPAI) is caused by viruses belonging to the genus *Alphainfluenzavirus*, species influenza A, and family *Orthomyxoviridae* [1]. Their low pathogenic avian influenza (LPAI) progenitors are found in avian aquatic reservoir species. Since 2005, repeated waves of introduction of an Asian origin HPAI H5Nx Goose/Guangdong (Gs/Gd) lineage have caused large numbers of mortality events and outbreaks among free-living wild birds, poultry, and other captive birds in Europe [2,3] and elsewhere. Like all avian influenza viruses (AIVs), this particular virus lineage undergoes mutation and genome exchange, leading to reassortants with different subtypes and genotypes and potentially new characteristics [4]. There is ample evidence of migratory wild birds acting as a major source of infection for poultry [5,6,7].

In Sweden, the first cases of HPAI (H5N1) were diagnosed in February to April 2006 in wild birds of nine different species, a mink, and a mallard on a mixed-species game bird farm [8]. Despite ongoing annual surveillance in wild birds in the following years, no additional HPAI positive cases were detected until 2015, when H5N8 (clade 2.3.4.4a) was isolated from two mute swans (*Cygnus olor*) in central Stockholm [9]. In the following season (2016–2017), 56 out of 273 sampled wild birds along the eastern coast of Sweden were diagnosed with HPAI H5N8 (clade 2.3.4.4b) [10]. Cases involved 15 species of waterfowl, raptors, and corvids. Moreover, for the first time, outbreaks were diagnosed in poultry on two commercial laying hen farms and in four hobby flocks with chickens and Muscovy ducks [10,11]. No signs of secondary spread to other farms were observed. During the following winter season in 2017–2018, one outbreak caused by H5N6 was confirmed in a hobby poultry flock, and a small number of cases in wild raptors (*N* = 14) were also found. During the influenza season of 2020–2021 (1 October 2020–30 September 2021), a large number of disease outbreaks were diagnosed across Europe, with 1385 documented outbreaks reported from poultry and other captive birds and 2408 wild bird reports [12]. As in the rest of Europe, the 2020–2021 influenza season was the hitherto most severe in Sweden and is the focus of this paper.

Sweden has a relatively small and low-density poultry industry compared to continental Europe. In June 2020, the number of laying hens and broiler chickens was estimated to be 8.4 million and 10.8 million birds, respectively [13]. In 2021, the number of laying hens in Sweden corresponded to 2.3% of the European Union population (10th place among the 27 European Union member states) [14]. In total, 112.4 million broiler chickens and 519,000 meat turkeys were slaughtered in Sweden in 2021 (rejected chickens not included) [15]. As a comparison, Sweden contributed 1.2% of the poultry meat produced in the EU in 2019 [15,16]. In contrast to some other European countries, the number of commercially raised ducks and geese was low with 12,000–13,000 birds slaughtered in 2020 of each species (pers. comm. Ingrid Medin, Swedish Food Agency, Uppsala, Sweden, 14 January 2021).

The Swedish avifauna includes around 250 reproducing species of wild birds, of which a majority are migratory, and an estimated number of 70 million breeding pairs [17]. Standardised surveys show that some wild species are decreasing in numbers while others are increasing [18]. One such example is that the number of wintering geese today is around eight times higher compared to the beginning of the 1980s [18].

The ever more frequent HPAI incursions during winter seasons in Europe are a severe threat to the poultry industry and this situation is not expected to improve in coming years. Even farms with a high level of biosecurity have been affected and there are still significant knowledge gaps regarding the wild bird/poultry interface, especially regarding how poultry can be protected. To this end, a broader perspective on the epidemiology and virology associated with these outbreaks is needed. The aim of this paper was to describe the 2020–2021 influenza season in Sweden and thereby contribute new aspects on the alarming HPAI situation in wild birds, poultry, and other captive birds.

## 2. Materials and Methods

### 2.1. HPAI Surveillance

Surveillance data from wild birds for analysis were retrieved from the National Veterinary Institute Uppsala, Sweden (SVA), database and analysed. Wild birds were sampled for AIV as part of passive surveillance of birds submitted for *post-mortem* examination to SVA. Members of the public reported dead or diseased wild birds through a web application and some of these birds were selected for necropsy and AIV diagnostics after a decision by a wildlife pathologist. Between October and December 2020, most reported birds were requested for examination. Thereafter, due to the high number of reported birds, species previously not confirmed positive in a given municipality during the preceding 30 days were selected for sampling and necropsy. This also applied to all reported bird species that are considered as state wildlife species i.e., vulnerable and protected species, which are mandatory to submit *post-mortem* to the Swedish Museum of Natural History, Stockholm, Sweden or to SVA. Small passerine birds and pigeons were excluded from AIV surveillance from March 2021 onwards. Thus, the number of confirmed HPAI-infected wild birds reported in this paper may represent single birds or separate events (outbreaks) involving any number of birds in a given place and time period. Active HPAI surveillance among wild birds was not carried out during the 2020–2021 season.

During most of the avian influenza season 2020–2021, HPAI was regulated by Council Directive 2005/94/EC [19]. According to the Directive and national legislation, it was mandatory for farmers, their staff, and other bird owners to notify symptoms suggestive of HPAI in poultry and other captive birds to a veterinarian. In turn, the veterinarian was obliged to investigate the symptoms and report to the veterinary authorities if HPAI was suspected. To further strengthen the passive clinical surveillance in poultry and other captive birds during the 2020–2021 influenza season, a multitude of awareness-raising activities were initiated targeting veterinary practitioners, poultry industry stakeholders, farmers, and owners of hobby flocks. The following symptoms were emphasized: Sudden onset of signs of disease or mortality, drop in egg production, egg abnormalities (misshapen eggs and/or, shell-less eggs) and respiratory, gastrointestinal, or neurologic signs. Reports of suspected disease outbreaks were assessed by veterinary epidemiologists and poultry veterinarians at SVA and, if HPAI could not be dismissed, birds were requested for analysis after the decision by the Swedish Board of Agriculture. An HPAI outbreak was defined as a laboratory-confirmed HPAI diagnosis in a flock of poultry or other captive birds independent of its size and category. Stamping out was applied on farms with confirmed HPAI, and restriction zones were established in a 3 km (protection zone) and 10 km (surveillance zone) radius around outbreaks in commercial poultry flocks. Based on farm-specific risk assessments, a decision was made in each case whether individual flocks could be considered separate epidemiological units and thereby be exempt from euthanasia. All poultry farms within protection zones and a selection of farms in surveillance zones were visited by official veterinarians who performed clinical examinations and checked records of daily mortality, egg production, egg quality and feed and water consumption. If suspected clinical signs were noted, samples were obtained to rule out HPAI. Active surveillance for AIVs outside restriction zones was carried out among commercial poultry, including game pheasants and mallards, through sampling at slaughter or on farms. This was part of the mandatory joint surveillance activities carried out by all EU member states.

### 2.2. Sampling

From wild birds, cloacal and pharyngeal swabs were collected for PCR prior to necropsy. From poultry and other captive birds, liver, spleen, trachea/lung, oviduct, kidney, caecal tonsils, and brain were obtained from up to five dead or euthanised birds per flock for PCR.

### 2.3. PCR and Whole Genome Sequencing

Laboratory diagnostics for AIV were performed in a BSL3 laboratory. Viral RNA was extracted using the TANBead nucleic acid extraction kit on the Maelstrom 9600 platform (Taiwan Advanced Nanotech Inc., Taoyuan, Taiwan). Extracted RNA was tested with one-step real-time reverse transcriptase PCR (RRT-PCR) assays recommended by the European Reference Laboratory for Avian Influenza, Padua, Italy (EURL, ISZVE). Initially, samples were screened for the presence of influenza A by the M-gene RRT-PCR [20], and positive samples were subsequently tested with hemagglutinin (HA) gene-specific RRT-PCR for detection of H5 and H7 subtypes [21,22]. The neuraminidase (NA) subtypes of H5 positive samples were determined by using multiple oligonucleotide sets based on the Riems Influenza A Subtyping Assay (RITA) [23]. Pathogenicity determination was performed either by Sanger sequencing across the HA proteolytic cleavage site using the PCR fragment generated with the previously reported conventional RT-PCR assay [24] or an H5-specific pathotyping RRT-PCR assay [25].

Whole-genome sequencing of the influenza A virus genome was performed using a modified multi-segment whole genome amplification protocol [26]. Briefly, SuperScript™ IV One-Step RT-PCR System with Platinum™ Taq High Fidelity DNA Polymerase (Invitrogen, Waltham, MA, USA) was used to amplify the whole genome. cDNA purification was carried out using AMPure beads (Beckman Coulter, Indianapolis, IN, USA), and the product was quantified with Qubit™ DNA HS Assay (Thermo Fisher Scientific, Waltham, MA, USA). Library preparation was performed using the NEXTERA-XT kit (Illumina Inc., San Diego, CA, USA) according to the manufacturer’s instructions. The Agilent 2100 Bioanalyzer (Agilent Technologies, Santa Clara, CA, USA) was used to assess the quality of the obtained libraries. The libraries were sequenced on an Illumina MiSeq instrument (Illumina Inc., San Diego, CA, USA), using a Miseq Reagent Kit v3 in a 600-cycle paired-end run. Quality analysis and filtering of the raw reads were performed by CLC genomics workbench 21.0.1 (CLC bio, Aarhus, Denmark). Complete genomes were generated through a reference-based approach. The consensus nucleotide sequence was compared to the NCBI GenBank database, using the BLAST algorithm Search Tool (https://blast.ncbi.nlm.nih.gov/Blast.cgi, accessed on 21 September 2021, version BLAST+ 2.13.0). Phylogenetic trees were constructed by using the sequence of each individual gene segment separately together with relevant nucleotide sequences available in GenBank. Blast homology searches were used to retrieve the top hundred homologous sequences from GenBank. The maximum likelihood (ML) method implemented in the MEGA11.0.10: Molecular Evolutionary Genetics Analysis version 11.0.26 [27] tool was used. The robustness of the ML trees was statistically evaluated by bootstrap analysis with 2000 replicates.

### 2.4. Epidemiological Investigation

Tracing interviews with all poultry farmers were carried out by official veterinarians appointed by the Swedish Board of Agriculture, and reports were made available to SVA. Information on the time of onset of symptoms to identify relevant periods for contact tracing, direct and indirect contacts, presence of wild birds and biosecurity aspects were collected and analysed to identify contact farms and possible routes of introduction. Additional epidemiological information on identified contacts was collected by veterinary officers at the Swedish Board of Agriculture and shared with SVA. If contact farms were identified, samples were taken to rule out HPAI. To collect further epidemiological information, outbreak farms were visited by epidemiologists from SVA in four cases and telephone or online interviews were conducted in four cases. As the outbreaks coincided with the SARS-CoV-2 pandemic, opportunities to visit HPAI outbreak farms by central veterinary staff were limited due to travel restrictions.

## 3. Results

### 3.1. Outbreaks

**Figure 1:**
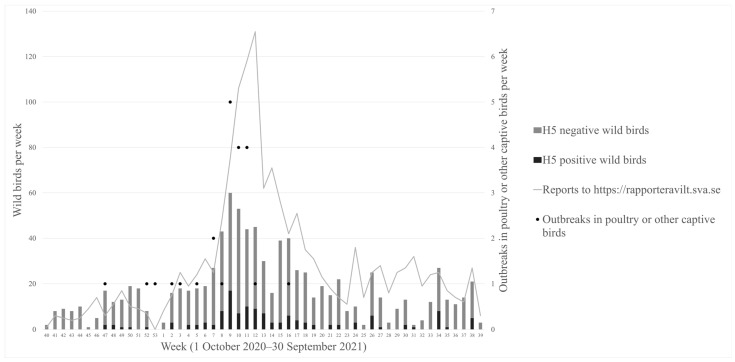
Weekly reports of diseased/dead wild birds to the National Veterinary Institute, number of wild birds sampled and H5 positive or negative within the avian influenza surveillance program and number of outbreaks in poultry and other captive birds during the influenza season 2020–2021 in Sweden. Note different scales on left and right X-axes.

During the 2020–2021 HPAI season (between 1 October 2020 and 30 September 2021), more than 1500 dead and diseased wild birds across Sweden were reported to SVA by members of the public. Avian influenza A virus (H5) was detected in 130 out of 811 tested wild birds. Tested birds comprised 94 species and HPAIV was detected in 24 of these, mostly in waterfowl and raptors (Table 1). A list of all sampled wild bird species is shown in Table S1. The first case of the season in wild birds was diagnosed on 26 November 2020 and the last case on 27 September 2021 (Figure 1), but cases continued to appear after this date (allocated to the next season). One infected eagle owl was found dead already on 30 October 2020 but was not submitted until 28 January 2021.

During the same period, 65 suspected outbreaks in poultry and other captive birds were investigated. The first poultry HPAI outbreak was diagnosed on 14 November 2020 and the last one on 20 April 2021. The most common clinical complaint reported by farmers/bird owners and referring veterinarians was a sudden increase in flock mortality. Depression, inappetence, decreased or increased water intake, respiratory distress, neurologic signs, inactivity and loss of vocalisation, wet litter, faecal staining of eggshells and soft-shelled eggs were reported from occasional flocks. In total, outbreaks were confirmed on 24 farms (Table 2 and Table 3, case ID numbers are shown in Table 3). One large farm complex was counted as two separate outbreaks in this paper as it involved laying hens and pullets, respectively, but it was officially reported as a single outbreak. The outbreaks were diagnosed on 15 commercial poultry farms, a game farm with pheasants, in seven hobby flocks and in a zoological collection with poultry. Five out of the seven hobby flocks and the zoo collection comprised mixed gallinaceous and anseriform species, while chickens were the only species in the remaining two hobby flocks. Multiple flocks were present on some commercial farms, and in most cases, all flocks were euthanised upon primary diagnosis. On two large farms, the initial risk assessment following primary diagnosis suggested that the barns could be considered as separate epidemiological units, thus it was decided to cull infected flocks only and keep the other flocks under restrictions and strict clinical surveillance. This was unsuccessful as transmission soon occurred to noninfected flocks on the same farm, and all birds had to be euthanised. In total, more than 2.2 million poultry died or were euthanised as a consequence of the outbreaks (Table 2).

**Figure 2:**
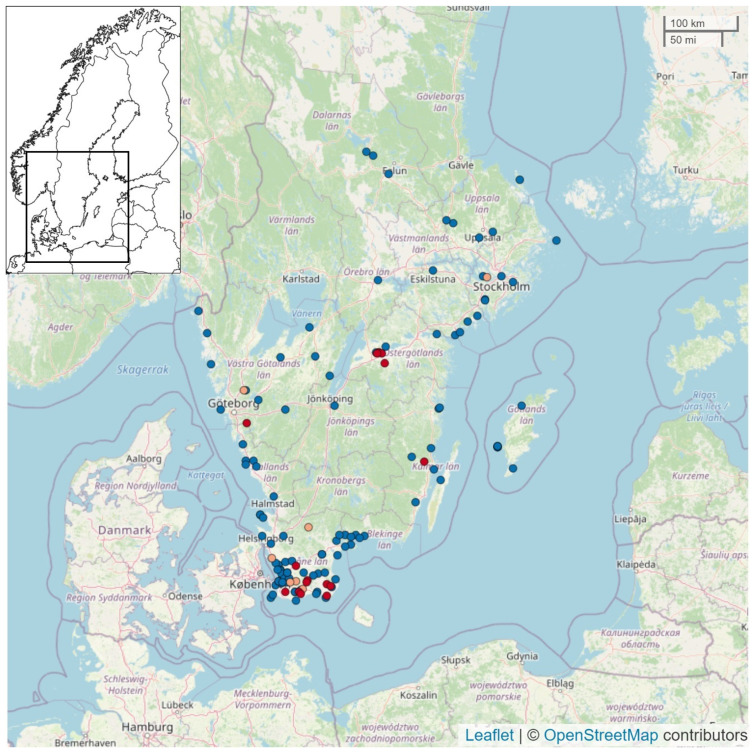
Geographic locations of reported cases of HPAI in poultry (red), other captive birds (yellow) and wild birds (blue) during the influenza season 2020–2021 in Sweden. Wild bird locations represent single or multiple infected birds. Maps generated with data from OpenStreetMap^®^, https://www.openstreetmap.org/copyright, accessed on 27 May 2022, Source: European Animal Disease Information System [28]. Access date 25 May 2022.

**Figure 3:**
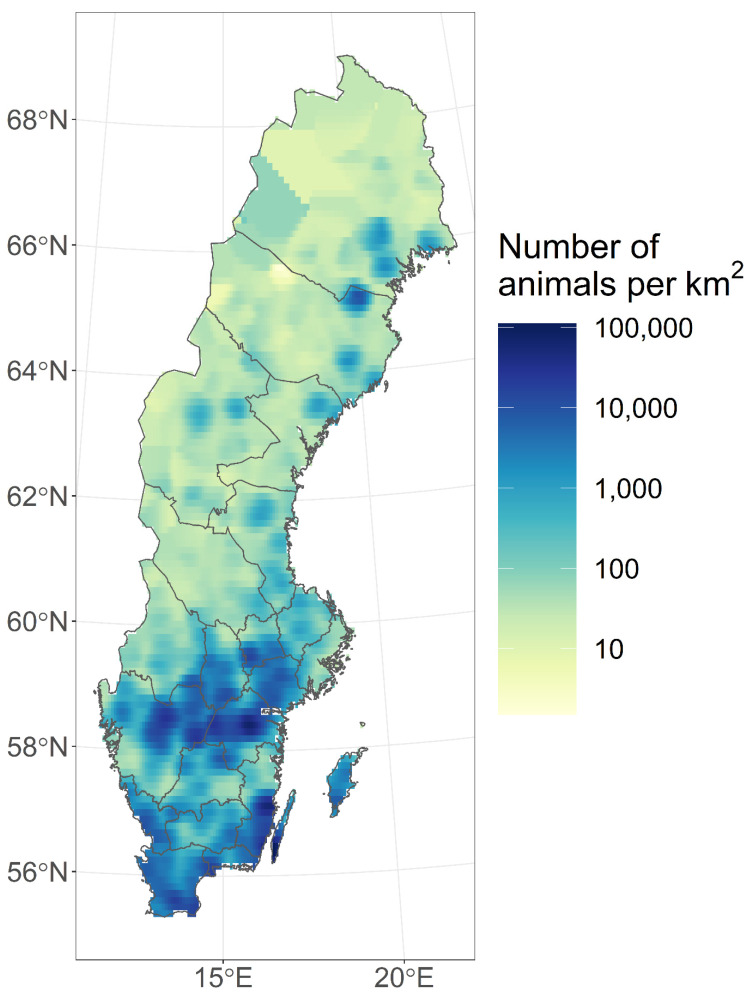
Map of Sweden showing domestic poultry density (number per square kilometre, colours in key). Map was created using kernel smoothing with a 10,000 m smoothing bandwidth over the value (animal count) at each point (farm identity (PPN)) location. Source: Swedish poultry register, Swedish Board of Agriculture.

As can be seen from Figure 1 and Figure 2, there appeared to be a geographic and temporal association between cases in wild birds and poultry. Most cases were found in the southern third of Sweden along the coast or in close proximity to lakes and other bodies of water. Outbreaks in poultry were also often located in areas with more-dense poultry populations (Figure 3). There was a peak in the number of infected wild birds and poultry outbreaks in February and March 2021. A novel feature of this HPAI season was that it lasted over the summer into September in wild birds.

### 3.2. Virology and Phylogeny

Extensive circulation of HPAI H5 viruses among multiple species of wild birds resulted in the detection of 130 H5-positive samples out of the 811 sampled birds. These included H5N1 (*n* = 7), H5N4 (*n* = 2), H5N5 (*n* = 21), H5N8 (*n* = 91) and H5Nx (*n* = 9). In total, 121 out of the 130 samples were confirmed as highly pathogenic and for the remaining nine samples pathogenicity was inconclusive. HPAI was detected in seven poultry hobby flocks (H5N5 (*n* = 3) and H5N8 (*n* = 4)), one zoo collection (H5N8 (*n* = 1)), one game bird farm (H5N8 (*n* = 1)) and fifteen commercial poultry farms (H5N5 (*n* = 3) and H5N8 (*n* = 12)). Almost all detected H5 viruses were identified as HPAIV phenotype based on the presence of multiple basic amino acids at the HA proteolytic cleavage site (PLREKRRKR*GLF (*n* = 141), PLKEKRRKR*GLF (*n* = 1)) and PLRGKRRKR*GLF (*n* = 3).

**Figure 4:**
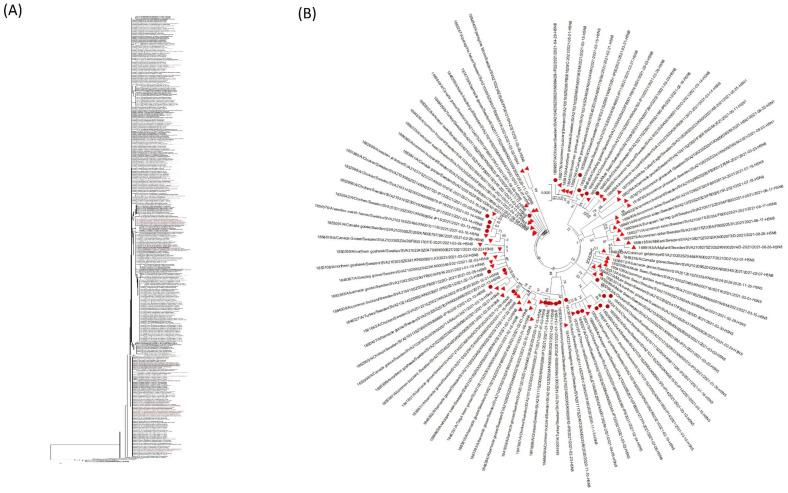

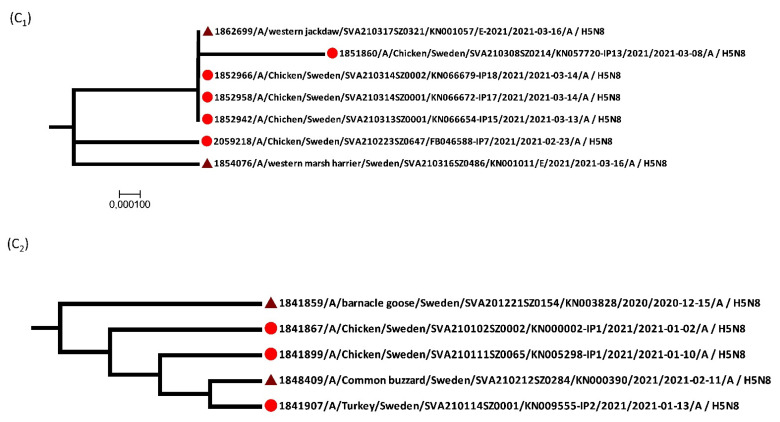
Maximum-likelihood phylogenetic tree for the haemagglutinin gene. (**A**) The phylogenetic tree was constructed with H5Nx viruses reported in Europe (866) from 1 October 2020–30 September 2021. Viruses detected in Sweden are indicated by red text. (**B**) The phylogenetic tree was constructed with H5Nx viruses detected in Sweden (91) from 1 October 2020 to 30 September 2021. Red solid triangles indicate the samples from the wild birds and red solid circles are indicating the samples from poultry and other captive birds. (**C_1_**,**C_2_**) Genetic relationship between sequences of detected viruses in the cluster of farms IP1 and IP2 and in the cluster of neighbouring farms IP13, IP15, IP17 and IP18. Bootstrap values (2000 replicates) >70% are displayed at the branch nodes. The scale bar indicates the number of nucleotide substitutions per site.

**Figure 5:**
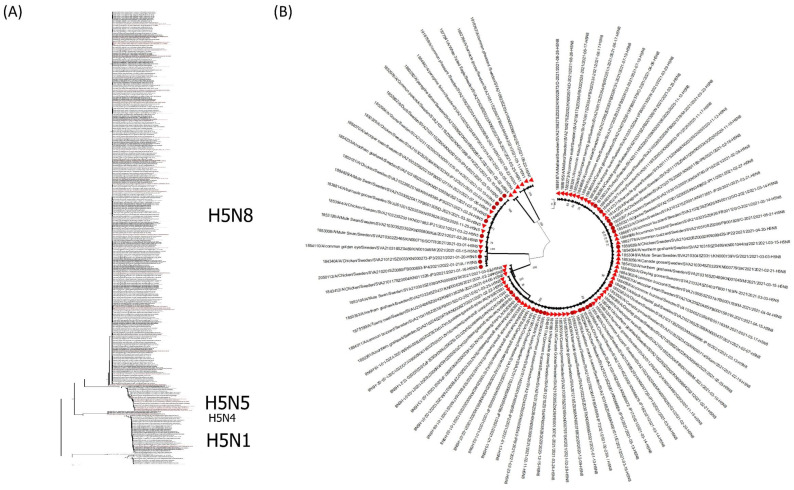

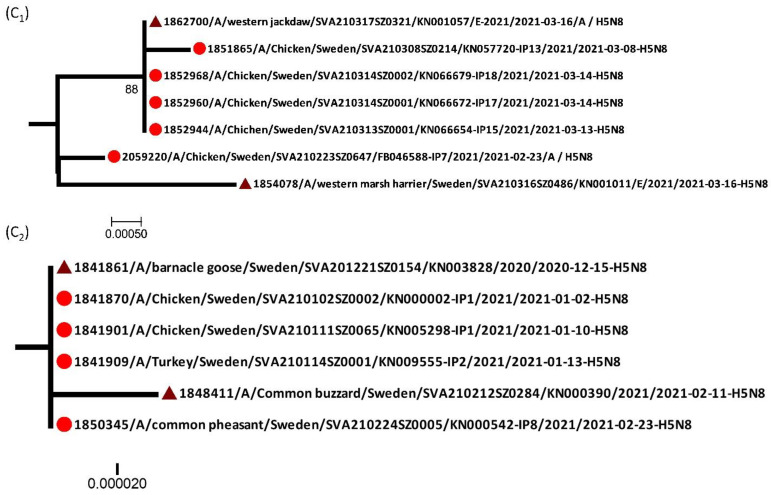
Maximum-likelihood phylogenetic tree for the NA gene. (**A**) The phylogenetic tree was constructed with H5Nx viruses reported in Europe (*n* = 813) during the influenza season 2020–2021. Viruses detected in Sweden are indicated by red text. (**B**) The phylogenetic tree was constructed with H5Nx viruses detected in Sweden (*n* = 91) during the influenza season 2020–2021. Red solid triangles indicate the samples from the wild birds and red solid circles are indicating the samples from poultry and captive birds. (**C_1_**,**C_2_**) Genetic relationship between sequences of detected viruses in the cluster of farms IP1 and IP2 and in the cluster of neighbouring farms IP13, IP15, IP17 and IP18. Bootstrap values (2000 replicates) >70% are displayed at the branch nodes. The scale bar indicates the number of nucleotide substitutions per site.

To investigate the genetic relationship between the HPAI viruses detected in poultry, other captive birds and wild birds in Sweden, the complete genome sequences of 91 viruses (poultry and captive birds *n* = 28, wild birds *n* = 64) were determined. The viral sequences generated for this paper are publicly available in the GISAID database (http://platform.gisaid.org; accessed on 4 January 2022); the accession numbers of the representative viruses are listed in Table S2. The topology of the HA phylogenetic tree showed that the Swedish H5Nx viruses belonged to clade 2.3.4.4b of H5 Gs/Gd lineage. This clade represents the dominating genetic group of the Gs/Gd H5 HPAI in western Europe in recent years. The phylogenetic analysis of the HA and NA genes suggested several independent introductions from wild birds to poultry (Figure 4 and Figure 5) except for a close genetic relationship between the isolates from IP1 and IP2 (Figure 4 and Figure 5), and between the isolates from IP13, IP15, IP17 and IP18 (Figure 4 and Figure 5)—see Section 3.3 and Table 3. This suggested either secondary spread or introduction from a common source in the wild bird population. There were limited surveillance data from wild birds in these areas during this period of time. The results also suggest persistent circulation of genetically related HPAI H5N8 and H5N5 viruses during the 2020–2021 influenza season and the incursion of novel HPAI H5N1 and H5N4 in the spring of 2021.

### 3.3. Epidemiological Investigation

As there was a nationwide housing order for all free-ranging commercial poultry in place prior to the outbreak the likelihood of direct contact with wild birds was low. The epidemiological investigation revealed no direct contacts between wild birds and poultry on the commercial outbreak farms. Based on a combination of epidemiological investigations on outbreak farms and phylogenetic data, it was concluded that most of the introductions of HPAI to commercial poultry were likely caused by indirect contact with wild birds (Figure 4 and Figure 5). Some of the non-commercial farms did keep poultry with outdoor access and direct contact with wild birds could not be ruled out in these cases.

Occasional weak points in biosecurity routines were identified on some of the commercial poultry farms. Examples reported from official veterinarians were rodents and rodent droppings within barns, poorly maintained buildings, uncovered ventilation outlets, and synanthropic wild birds (e.g., corvids, pigeons, and small passerine birds) in and around barns. Ponds or other stagnant water near poultry barns that could attract wild waterfowl were also observed on some farms. Compliance with biosecurity protocols could not be assessed. Language barriers were identified on some farms, which could potentially affect implementation of biosecurity protocols, but measures to overcome the barriers, e.g., translations of biosecurity instructions and bilingual supervisors were reported to be in place.

Presence of wild waterfowl, predominantly geese, in close proximity to the poultry farms was reported by at least seven commercial farmers. Some farmers were specific regarding species and numbers of wild birds, but others were not. A definite route of introduction from wild birds could not be identified on any of the farms. Hygiene barriers, which on some farms included showering in, were reported to be present and used on all commercial farms, but information on these aspects was lacking from hobby flocks.

No spread between poultry farms by direct or indirect contact was confirmed. Virus transmission between neighbouring flocks on the same farm was, however, suspected in several cases. Among these were the two farms where all flocks were not euthanised immediately following the initial virus detection. The epidemiological investigation concluded that airborne virus spread may have occurred between epidemiological units on these two farms following carbon dioxide (CO_2_) culling when the ventilation was switched on and the doors were opened. This assumption was based on the time of onset of symptoms, location of the barns, registered wind direction, and time of culling and emptying of barns with confirmed positive flocks. Epidemiological investigations of the spatiotemporal cluster of four outbreaks on laying hen farms within a six-kilometre radius (see Section 3.2) were inconclusive. No common direct or indirect contacts were identified. Airborne spread between these farms could not be ruled out, but undisclosed indirect contact or a common source within the wild bird population were other possibilities.

## 4. Discussion

In Europe, including Sweden, the number of species affected and number of diagnosed cases were far greater during the 2020–2021 HPAI season in wild birds, poultry and other captive birds compared to previous seasons [29]. The virus was introduced to Sweden through migratory wild waterfowl, a phenomenon seen previously but never before to this degree. Sweden was severely affected by HPAI in relation to the size of the poultry population. The number of birds that died or were euthanised due to outbreaks in 2020–2021 was an estimated ten percent of the poultry population (chickens on farms with >1000 birds) present in Sweden according to the last census conducted prior to the outbreak [13]. The number of birds that died or were euthanised due to HPAI in Sweden constituted approximately nine percent of the total number reported in Europe during the 2020–2021 season [28], while Sweden’s layer population constituted only 2.3% and poultry meat only 1.2% of the total EU production. The economic impact and the negative effects on the welfare of poultry and other captive birds were detrimental. The outbreaks also had substantial secondary effects such as a shortage of both hatching eggs and table eggs as well as pullets. The major reason was that all chickens on several key farms with laying hens, layer pullets and parent stock for production of broiler hatching eggs had to be euthanised. As a consequence, for the first time in some years, there was a dip in the trend towards an increased number of laying hens with 24% fewer laying hens in June 2021 compared to 12 months earlier [13,30]. This illustrates the vulnerability of the poultry value chain. The shortages had to be alleviated by international trade of hatching eggs with resulting challenges to avoid introduction of pathogens normally not present within the Swedish poultry population.

Despite numerous confirmed HPAI-infected wild birds during the 2020–2021 season, the true number of affected birds was most likely much higher, and the true range of affected species as well as geographic distribution were likely underestimated. The HPAI surveillance in wild birds in Sweden depends on the detection and voluntary reporting of diseased or dead birds by the general public. This in turn depends on local human population density and the frequency of visits by people along flyways, in coastal areas, wetlands and other localities where susceptible wild birds aggregate and breed, as well as the level of knowledge about the reporting system and the willingness to report. Large conspicuous birds such as swans and large raptors attract attention and may therefore be overrepresented, and each bird may be reported more than once. Thus, the relative numbers of infected birds of different species are not necessarily representative of the disease situation. It has not been investigated how well the reporting reflects true mortality, but based on the above, a large variation is likely to be present. Further, the selection of wild birds for necropsy and sampling, as described above (Section 2.1), most likely influenced the species spectrum and relative numbers of birds per species and geographical area examined. One example is that there were large numbers of dead and diseased barnacle geese reported from local outbreaks, but only a few birds were selected for sampling and were included as confirmed cases. In contrast, a small number of infected white-tailed eagles were diagnosed with HPAI despite the large number of sampled birds (4 out of 90, Table 1). This can be explained by the fact that this species is mandatory to submit for examination (state wildlife species) regardless of suspected cause of death, and all of them were analysed.

The long-term effects of HPAI infections in wild bird populations are still largely unknown. The effect on different species will probably vary depending on factors such as susceptibility to the virus, reproductive capacity, and original population size. The number of dead peregrine falcons (a state wildlife species) reported and examined during the 2020–2021 season increased by more than 100% compared to the same time period one and two years previously (data from the SVA wildlife disease surveillance, results not shown). Approximately 50% of the examined peregrine falcons had died due to HPAI (Table 1). This suggests a substantial effect on the wild peregrine falcon population, which is a near-threatened species in Sweden [31]. Another example was the eagle owl, which is classified as a vulnerable species [31]. Approximately 40% of the examined dead eagle owls were diagnosed with HPAI (Table 1) despite that they mainly prey on small rodents. However, they are also known to hunt larger prey, including waterfowl [32].

During the 2020–2021 HPAI season, there was a spatiotemporal clustering among observed cases in poultry and wild birds. The location of cases was predominantly in the southern third of Sweden, close to the coast or lakes or other bodies of water and near migratory pathways for wild birds (Figure 2). The areas with outbreaks in poultry coincided to a large extent with a higher density of poultry (Figure 3). Furthermore, there was a temporal clustering of cases with a peak in the number of positive cases in February and March 2021 (Figure 1), which coincided with a peak in cases in the rest of Europe [29]. Another temporal pattern that was noted in Sweden and other European countries was that the virus persisted throughout the summer in wild birds. In two separate geographic locations there were events with high mortality in wild birds in mallards in August and in pheasants in September. This was attributed to virus persistence due to the high occurrence of HPAI in the spring of 2021, which has not been observed in previous seasons.

As in previous outbreak seasons, it appeared that indirect or direct contact with infected wild birds was the main source of infection in poultry and captive birds. Several separate primary introductions are likely to have caused the outbreaks. The sequenced viruses detected in poultry and captive birds clustered phylogenetically with the sequences detected in wild birds (Figure 4). Reports of HPAI outbreaks in other European countries also describe wild birds as the main source of introduction of HPAI to poultry [33,34]. Except for non-commercial flocks, all poultry were confined indoors during the 2020–2021 season as there was a nationwide housing order enforced in Sweden. Keeping poultry indoors has been shown to reduce the risk of introduction of LPAI virus [35] and an expert elicitation has proposed that the same is true for HPAIV [36]. Thus, the housing order is likely to have reduced the risk and thus the number of outbreaks during the 2020–2021 season.

Although breaches in biosecurity and possible routes of introduction from wild birds were identified in several cases, a definite route and mechanism of transmission could not be established in any of the outbreaks on commercial poultry farms. The epidemiological investigations identified the need for further studies to investigate HPAI risk factors in Sweden, specifically regarding biosecurity. Previously, biosecurity practices in broiler production in Sweden have been investigated in relation to risk factors for presence of *Campylobacter* spp. [37]. Further, a questionnaire study to investigate biosecurity routines on Swedish laying hen farms in 2017 reported little variation between farms [38]. It could be argued that the questionnaire format may not be ideal for identifying true differences and that biosecurity compliance also needs to be evaluated. However, comparative studies including different production types, and studies with specific focus on HPAI are lacking in the Swedish context. To overcome limitations with questionnaire studies, an approach including on-farm observations may be better to identify true variations.

Several countries in Europe confirmed secondary spread of HPAI between poultry farms during the 2020–2021 season [29,34]. From the epidemiological investigations and the phylogenetic analysis, secondary spread between farms through direct or indirect contact was not confirmed in Sweden. However, there was some uncertainty regarding the outbreaks of IP1 and IP2 and the cluster of neighbouring farms (IP13, IP15, IP17 and IP18) as the phylogenetic analysis confirmed a close genetic similarity within the two groups (Figure 4). Although the investigations didn’t identify any common contacts, undisclosed indirect contacts could not be ruled out. A common source within the wild bird population was a possibility and another possible explanation was that the cluster may have been a result of airborne transmission (see below). A factor that contributed to the uncertainty was the limited surveillance data available from wild birds in these areas during this time period. The biosecurity routines in combination with control measures in response to outbreaks appear to have been sufficient to prevent spread through direct and indirect contact between commercial farms. A contributing factor minimising the risk of spread between farms was the relatively low density of poultry farms, reducing risk of local spread. Also, the poultry population in Sweden consists mainly of chickens and the population of ducks and geese is very small. Many cases of secondary spread reported from other European countries have involved anseriform birds [29,34,39,40]. Another factor that may mitigate the risk of spread between farms is the long history of prevention of *Salmonella* spp., *Campylobacter* spp. and avian paramyxovirus type 1 (Newcastle disease) in Sweden. Sweden currently is a non-vaccinating country as regards Newcastle disease, so biosecurity is the only available tool to prevent outbreaks. The main focus of the biosecurity programs in place has been to prevent introduction of these microorganisms. Preventive tools and measures have not been primarily designed to avoid indirect contact with wild waterfowl that periodically are found in large numbers on fields near barns. Rather, biosecurity measures have focused on keeping out synanthropic wild birds from barns and from access to feed and bedding material. As described, presence of wild waterfowl in close proximity to commercial poultry farms was reported in approximately half of the outbreaks. The fact that established farms in many instances are located near the coast or bodies of water cannot be easily overcome, but measures are needed to mitigate risks and adapt to the new HPAI situation in wild waterfowl.

Another route of virus spread that may have affected the course of the Swedish 2020–2021 avian influenza season is airborne transmission. Spread between barns on the same farm did occur, and airborne spread was considered likely in some cases given wind direction and the location of the separate barns. Airborne spread between poultry farms was also suspected in some cases but could neither be confirmed nor ruled out. Epidemiological investigations of a spatiotemporal cluster of four outbreaks in laying hen farms within a six-kilometre radius have so far been inconclusive and airborne transmission was one of several hypotheses. Mathematical dispersal modelling [41], including factors such as probable date of introduction, topography, and prevailing wind and weather conditions could potentially be used as a tool to estimate the likelihood that spread was in fact airborne. Other studies have suggested occurrence of airborne spread of HPAI [42,43,44], and it has also been suggested that the risk of airborne spread by dust and aerosols may increase at the time of culling and disposal of carcasses [45].

Several categories of poultry experienced outbreaks during the 2020–2021 HPAI season in Sweden. It appears however that there were more outbreaks affecting turkey flocks compared to broilers, considering the relative size of the population and number of farms. Experimental studies have suggested that turkeys are more susceptible to HPAI viruses than other poultry species [46]. Also, HPAI outbreaks are fewer in broilers than in some other poultry species and categories in Europe [47]. This may be the result of a variety of factors such as genetics [48], husbandry routines, and biosecurity levels. In other countries, duck and/or geese production has been severely affected by HPAI [34,39]. In Sweden, no cases of HPAI were confirmed in commercial anseriform poultry, but as discussed above, this production is very limited. However, among affected non-commercial flocks, a majority were mixed anseriform and gallinaceous poultry, which is a known risk factor for introduction of HPAIV.

Surveillance in wild birds can serve as an early warning system for the risk of introduction of HPAI in poultry. However, during the 2020–2021 season, and in contrast to the HPAI season in 2016–2017, the first poultry outbreak was diagnosed prior to detection in wild birds in Sweden. In addition, in five out of six counties with outbreaks, the first case in the county was in poultry or other captive birds and there was no early warning through wild bird surveillance at regional level. As described, the continuous wild bird surveillance in Sweden is passive and dependent on the general public reporting and submitting dead birds. Active surveillance is often project-based with a delay between collection and analysis of samples. While there are advantages involving the general public in surveillance, there are also limitations when it comes to coverage (both in space and time) and keeping the public alert and aware that reports are of interest. Additional and more systematic observations of morbidity, mortality, or targeted active surveillance of HPAI in susceptible wild bird populations in areas with high poultry density areas relevant in relation to migration routes may contribute to earlier detection, which could be used to raise alertness among poultry farmers and other bird owners. Targeted active surveillance has the advantage of also capturing HPAIV in wild bird species that may be asymptomatic carriers [49]. In addition to surveillance activities, further work to identify risk areas in Sweden based on historical HPAI cases, land use, migratory flyways and other risk factors could contribute to the identification of areas where poultry producers need to be extra cautious, even prior to detection of cases in wild birds. Targeted active surveillance in wild birds could also contribute to a better basis for such risk mapping.

Surveillance in wild birds serves as an early warning system and a way to monitor the current HPAIV situation. However, more knowledge is needed on which wild bird species are key in the AIV transmission to poultry in Northern Europe. Targeted active sampling of wild birds near poultry farms may contribute to the understanding of the epidemiological significance of different groups of wild birds in relation to virus transmission between poultry and wild birds in both directions. The sampling strategy applied in Sweden during the 2020–2021 season would need to be revised to be able to obtain this type of information.

The quality of the reports from farmers on presence of wild birds on their farm varied. On one farm there were diverging views among employees when describing numbers of wild birds and locations on the farm where birds were normally observed, which illustrated the challenges of assessing wild bird presence and possible routes of introduction through direct or indirect contacts. Wild bird presence on poultry farms has been studied in other countries using systematic observation or cameras [50,51]. A Swedish study in 2007 [52] used a questionnaire approach, thus facing the limitations mentioned above with farmer observations. Moreover, the waterfowl population has changed significantly in Sweden [18]. Further studies are needed to increase the knowledge of wild bird presence on Swedish poultry farms including factors that may attract or deter wild birds. Information could be gained through observations of wild birds or observation of traces indicative of possible indirect contact with wild birds, e.g., wild bird droppings in feed. Such knowledge could be used to address risks for poultry in the future, as well as prevent spillover to wild birds in case of outbreaks on poultry farms.

## 5. Conclusions

The Swedish poultry industry was struck hard by HPAI in the 2020–2021 season, and the outbreaks have put focus on knowledge gaps that need to be addressed. Despite accumulating valuable knowledge at a global level, differences in the poultry industry and wild bird ecology call for the need of understanding the national situation. Focus should be on wild bird surveillance and wild bird presence on farms, biosecurity, risk factors for the introduction of HPAIV to poultry, and airborne spread.

## Figures and Tables

**Table 1:** Wild bird species infected with HPAI H5Nx (clade 2.3.4.4) in Sweden during the 2020–2021 season. The numbers represent infected birds (positive) and the total number of tested birds of each species. In nine birds, pathogenicity was inconclusive but was assumed to be high. Migratory birds are indicated with bold text.

Bird Species (English)	Bird Species (Latin)	Positive/Tested
**Barnacle goose**	*Branta leucopsis*	15/19
**Peregrine falcon** ^1^	*Falco peregrinus*	12/22
**Common buzzard**	*Buteo buteo*	12/36
Eagle owl ^1^	*Bubo bubo*	11/22
**Mute swan**	*Cygnus olor*	11/35
Northern goshawk	*Accipiter gentilis*	9/34
**Common eider**	*Somateria mollissima*	8/10
**Whooper swan**	*Cygnus cygnus*	7/16
**Canada goose**	*Branta canadensis*	7/14
White-tailed eagle ^1^	*Haliaeetus albicilla*	7/90
Mallard	*Anas platyrhynchos*	6/37
Common pheasant	*Phasianus colchicus*	5/13
**Greylag goose**	*Anser anser*	4/11
Herring gull	*Larus argentatus*	3/18
Tawny owl	*Strix aluco*	2/18
**Common goldeneye**	*Bucephala clangula*	2/3
**Eurasian oystercatcher**	*Haematopus ostralegus*	2/2
**Great white-fronted goose**	*Anser albifrons*	1/1
**Western marsh harrier** ^1^	*Circus aeruginosus*	1/1
Western jackdaw	*Corvus monedula*	1/31
Hooded crow	*Corvus cornix*	1/16
**Black-headed gull**	*Chroicocephalus ridibundus*	1/12
**Bean goose**	*Anser fabalis*	1/2
**Common kestrel** ^1^	*Falco tinnunculus*	1/16

^1^ State wildlife species.

**Table 2:** Summary of HPAI outbreaks in poultry and captive birds during the 2020–2021 season in Sweden.

Bird Category	No. of Farms			No. Susceptible Birds
	H5N8	H5N5	Total	
Laying hens (aviary, indoor)	2	1	3	1,250,000
Laying hens (organic)	3	1	4	85,000
Layer pullets	0	1	1	735,000
Broiler parents	2	0	2	138,000
Broilers (organic)	1	0	1	14,300
Turkeys (meat-type)	4	0	4	41,000
Game pheasants	1	0	1	500
Non-commercial poultry	4	3	7	450
Zoological collection (mixed poultry species)	1	0	1	38
**Total**	**18**	**6**	**24**	**2,264,288**

**Table 3:** HPAI H5Nx, clade 2.3.4.4.b outbreaks in poultry and captive birds during the 2020–2021 season in Sweden. Flock size and age refers to the first affected flock/flocks on outbreak farms.

No./Case ID	Poultry Category	No of Susceptible Animals on Farm	Initially Affected Flock		Date of Diagnosis	Subtype	County
			**Flock Size**	**Age**			
1 (1/2020)	Turkeys (meat-type)	5100	2000	12 weeks	14 November	H5N8	Skåne
2 (2/2020)	Hobby chickens	30	30	Adults	22 December	H5N8	Skåne
3 (IP1)	Broiler breeders (parents)	84,850	4300	28 weeks	3 January	H5N8	Skåne
4 (IP2)	Turkeys (meat-type)	2350	850	16/17 weeks	14 January	H5N8	Skåne
5 (IP3) ^1^	Laying hens (aviary, indoor)	1,200,000	85,000	42 weeks	18 January	H5N5	Kalmar
6 (IP4) ^1^	Layer pullets	735,000	245,000	16 weeks	1 February	H5N5	Kalmar
7 (IP5)	Turkeys (meat-type)	3500	3500	12 weeks	15 February	H5N8	Skåne
8 (IP6)	Hobby chickens and ducks	46	46	Adults	17 February	H5N8	Västra Götaland
9 (IP7)	Broiler (organic) ^2^	14,300	4880	60 d	24 February	H5N8	Östergötland
10 (IP8)	Game pheasants	470	470	Unknown	24 February	H5N8	Skåne
11 (IP9)	Hobby chickens	11	11	Adults	28 February	H5N5	Skåne
12 (IP10)	Hobby mixed species ^3^	263	263	Mixed ages	1 March	H5N8	Halland
13 (IP11)	Zoological collection ^4^	38	38	Adults	2 March	H5N8	Skåne
14 (IP12)	Laying hens (organic)	18,000	18,000	80 weeks	3 March	H5N5	Skåne
15 (IP13)	Laying hens (organic)	24,000	24,000	64 weeks	8 March	H5N8	Östergötland
16 (IP14)	Hobby mixed species ^5^	33	33	Adults	11 March	H5N5	Skåne
17 (IP15)	Laying hens (aviary, indoor)	33,000	33,000	53 weeks	13 March	H5N8	Östergötland
18 (IP16)	Broiler breeder parents	53,200	13,300	36 weeks	14 March	H5N8	Skåne
19 (IP17)	Laying hens (aviary, indoor)	21,000	21,000	22 weeks	15 March	H5N8	Östergötland
20 (IP18)	Laying hens (organic)	26,400	18,000/8400 ^6^	68 weeks	15 March	H5N8	Östergötland
21 (IP19)	Turkeys (meat-type)	30,000	2300/2300 ^6^	11/18 weeks	16 March	H5N8	Skåne
22 (IP20)	Hobby mixed species ^7^	63	63	Adults	21 March	H5N8	Skåne
23 (IP21)	Hobby chickens and ducks	14	14	Adults	23 March	H5N5	Stockholm
24 (IP22)	Laying hens (organic)	18,000	18,000	42 weeks	20 April	H5N8	Skåne

^1^ IP3 and IP4 are listed as separate outbreaks in this paper but were officially reported as one outbreak since they belonged to the same farm complex; ^2^ Slower-growing genotype; ^3^ Mixed Species: chickens, domestic ducks, and pigeons; ^4^ Mixed species: chickens and domestic geese; ^5^ Mixed species: peafowl, domestic ducks, and domestic geese; ^6^ Two flocks affected simultaneously; ^7^ Mixed species: chickens, domestic ducks, and domestic geese.

## Data Availability

Data is contained within the article or Supplementary Materials.

## Supplementary Materials

The following supporting information can be downloaded at: https://www.mdpi.com/article/10.3390/vetsci9070344/s1, Table S1: Wild bird species (94 species, *n* = 811 birds) sampled for avian influenza virus during the avian influenza season 2020–2021 in Sweden. Species was not identified in four sampled doves and one swan. ^1^ State wildlife species. Table S2: Clade 2.3.4.4b H5Nx highly pathogenic avian influenza viruses were used in the phylogenetic analysis. Nucleotide GISAID accession numbers for 91 H5Nx subtype viruses detected in Sweden during influenza season 2020–2021.
